# A New CuSe-TiO_2_-GO Ternary Nanocomposite: Realizing a High Capacitance and Voltage for an Advanced Hybrid Supercapacitor

**DOI:** 10.3390/nano13010123

**Published:** 2022-12-26

**Authors:** Muhammad Sajjad, Abdul Jabbar Khan, Sayed M. Eldin, Asma A. Alothman, Mohamed Ouladsmane, Patrizia Bocchetta, Waqas Ul Arifeen, Muhammad Sufyan Javed, Zhiyu Mao

**Affiliations:** 1 College of Chemistry and Life Sciences, Zhejiang Normal University, Jinhua 321004, China; 2 College of Chemistry and Chemical Engineering, Huanggang Normal University, Huangggang 438000, China; 3 Faculty of Engineering and Technology, Future University in Egypt, New Cairo 11835, Egypt; 4 Department of Chemistry, College of Science, King Saud University, Riyadh 11451, Saudi Arabia; 5 Dipartimento di Ingegneria dell’Innovazione, Università del Salento, Via Monteroni, 73100 Lecce, Italy; 6 School of Mechanical Engineering, Yeungnam University, Daehak-ro, Gyeongsan-si 38541, Gyeongbuk-do, Republic of Korea; 7 School of Physical Science and Technology, Lanzhou University, Lanzhou 730000, China

**Keywords:** CuSe, aqueous electrolyte, power density, energy density, impedance

## Abstract

A high capacitance and widened voltage frames for an aqueous supercapacitor system are challenging to realize simultaneously in an aqueous medium. The severe water splitting seriously restricts the narrow voltage of the aqueous electrolyte beyond 2 V. To overcome this limitation, herein, we proposed the facile wet-chemical synthesis of a new CuSe-TiO_2_-GO ternary nanocomposite for hybrid supercapacitors, thus boosting the specific energy up to some maximum extent. The capacitive charge storage mechanism of the CuSe-TiO_2_-GO ternary nanocomposite electrode was tested in an aqueous solution with 3 M KOH as the electrolyte in a three-cell mode assembly. The voltammogram analysis manifests good reversibility and a remarkable capacitive response at various currents and sweep rates, with a durable rate capability. At the same time, the discharge/charge platforms realize the most significant capacitance and a capacity of 920 F/g (153 mAh/g), supported by the impedance analysis with minimal resistances, ensuring the supply of electrolyte ion diffusion to the active host electrode interface. The built 2 V CuSe-TiO_2_-GO||AC-GO||KOH hybrid supercapacitor accomplished a significant capacitance of 175 F/g, high specific energy of 36 Wh/kg, superior specific power of 4781 W/kg, and extraordinary stability of 91.3% retention relative to the stable cycling performance. These merits pave a new way to build other ternary nanocomposites to achieve superior performance for energy storage devices.

## 1. Introduction

**Figure 1:**
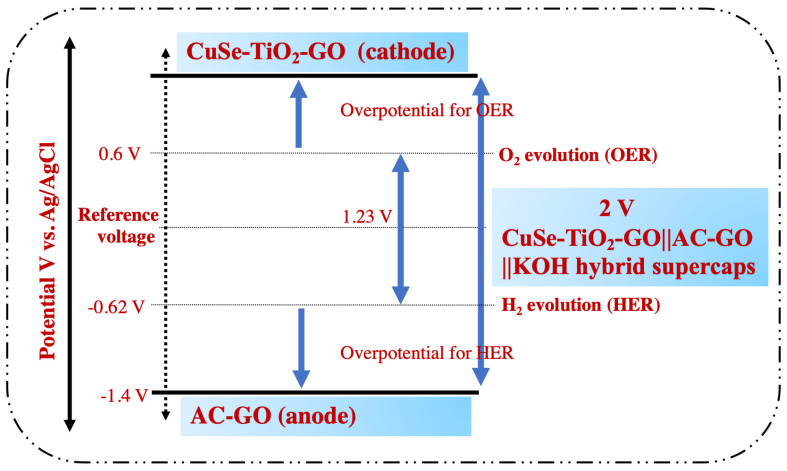
Schematic showing the expansion of the voltage region of the CuSe-TiO_2_-GO||AC-GO||KOH hybrid supercapacitor in an aqueous KOH electrolyte.

With the development of modern civilization, massive industrialization gives birth to huge carbon dioxide emissions, which cause serious environmental problems, such as global warming, air pollution, and the emission of toxic chemicals into the environment [1,2,3]. The poor energy density of supercapacitors can be optimized in two ways: either by increasing the capacitance or expanding the voltage frame of the symmetric/asymmetric/hybrid supercapacitors, as energy is related to the following expression [4,5]: E = ½ C × V^2^. The schematic, as given in Figure 1, presents the illustration of the expansion of the voltage gap in this work by the theoretical model. The electrolysis process paves a pivotal role in an aqueous supercapacitor. The voltage frame is limited due to the severe water splitting at 1.23 V^2^ [6,7,8], which can be boosted by optimizing the hydrogen evolution reaction (HER) that occurs on the positive electrode and the oxygen evolution reaction (OER) on the negative electrode in the KOH aqueous solution. This further realizes the optimized electrolyte/electrolyte interface. It is therefore expected that controlling the H^+^/OH^−^ generation effectively contributed to enhancing the final voltage limit of the potential electrodes [1,9], as given in Figure 1.

Graphene oxide (GO) anticipated a favorable position in the electrochemical energy domain due to its excellent chemical, optical, and mechanical properties [4,10,11]. The theoretical capacitance is ~550 F/g [12], and the performance in the supercapacitor is still far behind due to the severe agglomeration/restacking of different layers in applications coupled with the synthesis procedure. Mixing GO with metal oxides/selenides has been a hot topic for decades and has contributed substantially to defining the final charge storage performance of the obtained product material. Titanium dioxide, denoted as TiO_2_, is also utilized as an electrode for supercapacitors. Still, the free-standing electrode shows unsatisfactory charge storage abilities, such as low capacitance and determined conductivity during the charge storage process [13,14,15,16]. So far, metal oxide (V_2_O_5_, Co_3_O_4_, SnO_2_, MnO_2_, CdO, and Fe_2_O_3_)/GO [8,17,18,19,20,21] composites have been researched and reported to solve the stability of active electrodes and hydrophilicity-related drawbacks. Meanwhile, metal sulfides (FeS_2_, NiCo_2_S_4_, VS_2_, SnS_2_, WS_2_, MoS_2_, NiS, Ni_3_S_2_, CuS, and ZnS) [22,23,24,25,26,27,28,29] are also promising active materials for supercapacitors due to their high conductivity, suitable redox activities owing to multiple oxidation states, semiconducting behavior, and lower electronegativity. The main obstacles are the poor cycling lifespan due to the insulating behavior of sulfur, the thermodynamic instability, the volatility, and the expansion of sulfides-GO-based metal sulfide composites displayed an enhanced lifespan of pure metal sulfides owing to the synergistic effect. The capacitance and upper voltage limit have not been significantly enhanced so far; for instance, the rGO/TiO_2_/rGO ternary composite (114.5 F/g), with a potential of 0 to 0.8 V [30], GO/TiO_2_, with a capacitance of 73.43 mF/cm^2^ at a potential of 0–1 V [31], rGO-TiO_2_ composites 225 F/g at the current of 0.025 A/g [32], and a sandwich of a TiO_2_/rGO/TiO_2_ composite with 83.7 F/g [33] manifest that the performance needs to be further enhanced. To optimize the performance of the GO and TiO_2_ system, combining copper selenides (CuSe) [34,35,36] has recently been the hotspot and has substantial importance in selenide-based electrodes for electrochemical energy domains owing to their high conductivity, high theoretical capacity, several metallic states, and better energy storage performance in aqueous electrolytes (CuSe/CuSe_2_@GO with 192.8 F/g, with excellent stability until 10,000 cycles [37], the binderless electrode of CuSe_2_/Cu (1037 F/g with supreme power and energy densities) [38], and CuSe/NiSe [39] (1478/990 from 1 to 8 A/g) via the hydrothermal method) [40]. CuSe/Ni (OH)_2_ showed high stability and significant capacitance when utilized as an effective electrode for flexible supercapacitor applications [39]. Recently, our previous work demonstrated a heterostructured CuSe@TiO_2_ for a hybrid supercapacitor that boosts the low capacitance of CuSe to as high as 370 F/g from 225 F/g at a low current rate in an aqueous solution [34]. There is another report on the TiO_2_@CuSe nanocomposites from our group, with varying contents of CuSe and TiO_2_ in which the optimal electrode ZT-3 delivers a capacitance of 184 F/g and 135 at the currents of 2 to 9 A/g due to the synergy between the metal Ti and Cu elements, forming solid structural integrity and sustainability [41]. Inspired by these results and strategies, we follow the same materials, make a new CuSe-TiO_2_-GO ternary composite, and probe its electrochemical charge storage performance in a KOH solution. As stated above, we expected an excellent energy storage performance from this special heterostructure based on our previous experience.

This paper mainly provided insight into developing a new CuSe-TiO_2_-GO ternary composite for hybrid supercapacitor applications. The electrochemical properties reveal the prepared nanocomposite’s remarkable reversibility and redox activity in a KOH solution in the three-electrode assembly. A 36 Wh/kg specific energy was realized at 875 W/kg specific power, which expanded to its maximum value of 4781 W/kg after adding an optimized voltage of 2 V by constructing a CuSe-TiO_2_-GO||AC-GO|KOH hybrid supercapacitor.

## 2. Preparation of the Nanomaterials

### 2.1. Synthesis of CuSe

**Figure 2:**
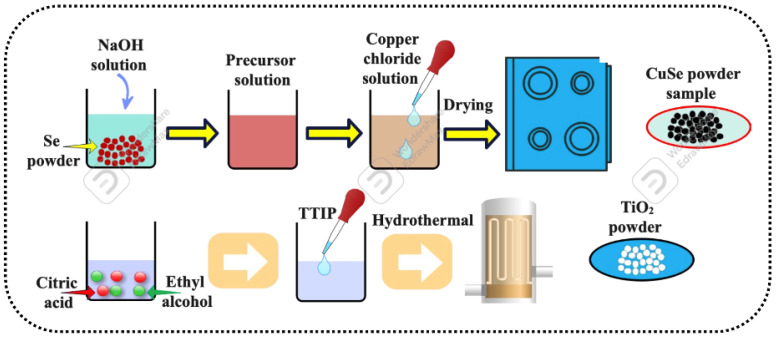
Illustration of the synthesis of the CuSe-TiO_2_ matrix.

To prepare the selenium alkaline aqueous solution, 4 g of elemental selenium (BDH) was dissolved in 12 M NaOH (Fisher Scientific, Beijing, China). As soon as the elemental selenium dissolved, the solution’s color changed to orange-red. After the elemental selenium was dissolved entirely, a Cu^2+^ solution made from CuCl_2_·2H_2_O (Fisher Scientific) was added drop by drop to the selenium alkaline aqueous solution and rapidly stirred. The produced black precipitate was centrifuged and rinsed with distilled water to remove the surplus alkaline solution. The powder precipitation was subsequently dried for 24 h in an oven at 343 K, as shown in Figure 2.

### 2.2. TiO_2_ Synthesis

The hydrothermal approach synthesized TiO_2_ nano-powders using titanium tetraisopropoxide, distilled water, ethyl alcohol, and citric acid as the starting ingredients. Under continuous magnetic stirring, titanium isopropoxide (TTIP) was added drop by drop to distilled water, ethyl alcohol, and citric acid. The mixture was stirred for another 2 h before being transferred to the stainless-steel autoclave with a Teflon lining. The sealed autoclave was heat-treated for three hours at 150 °C. The autoclave was withdrawn from the oven and cooled naturally to room temperature. The finished product was filtered and dried in an open environment, as depicted in Figure 2. 

### 2.3. Preparation of GO

GO was prepared according to the modified Hummer method [18,22]. In further detail, 108 mL of H_2_SO_4_, 12 mL of H_3_PO_4_, 5 g of graphite, 2.5 g of NaNO_3_, and 12 mL of H_3_PO_4_ were combined and agitated in an ice bath for 10 min. Afterward, 15 g of KMnO_4_ was added slowly while ensuring that the mixture’s temperature was maintained below 5 °C. The suspension was then stirred for 60 min and reacted for 2 h in an ice bath before being stirred once more for 60 min in a 40 °C water bath. The mixture was heated to a constant 98 °C for 60 min while water was continuously added. Additional deionized water was added until the suspension reached 400 mL in it. After 5 min, 15 mL of H_2_O_2_ was added. The result of the reaction was centrifuged and repeatedly rinsed with deionized water and a 5% HCl solution. At 60 °C, the material was finally dried.

### 2.4. Preparation of the CuSe-TiO_2_-GO Ternary Nanocomposite

By varying the wt.% ratios of TiO_2_ sheets and CuSe nanoparticles, CuSe-TiO_2_ nanocomposites were synthesized. Briefly, sonication was used to scatter 25% weight ratios of TiO_2_ (relative to CuSe) and CuSe nanoparticles in 40 mL methanol. This mixture was stirred for approximately thirty minutes before being thoroughly rinsed with DI water and ethanol. The product was subsequently dried at 60 °C; using the same process for CuSe-TiO_2_-GO nanocomposites, 50% weight ratios of CuSe-TiO_2_ and GO were achieved (relative to GO).

### 2.5. Characterization and Electrode Preparation

FESEM first examined the morphology and elemental analysis of the ternary nanocomposite with the TESCAN model MAIA-3 (Islamabad, Pakistan). The Raman spectroscopy is measured to the symmetry of different bonds in the CuSe-TiO_2_ and GO ternary composite by Model, Dongwoo Optron Co. Ltd (Islamabad, Pakistan). The crystal structure, facile preparation, and phase purity of the sample were detected by utilizing X-ray diffraction examination with the model number EQUINOX-3000 (Thermo Scientific, Islamabad, Pakistan), Cu Kα irradiation with a wavelength of 1.54 nm, and a step size of 0.01 at a 10 to 90º theta range.

Three- and two-cell modes were employed to probe the electrochemical properties of the novel CuSe-TiO_2_-GO ternary nanocomposite in an aqueous solution. Several electrochemical techniques were tested for functional electrochemistry studies at various scans and currents, such as impedance, charge/discharge platforms, and the cyclic voltammogram. A hybrid supercapacitor was assembled with AC-GO as an anode and CuSe-TiO_2_-GO ternary nanocomposite as the anode in a sandwich configuration using aqueous KOH as the electrolyte.

## 3. Results and Discussion

**Figure 3:**
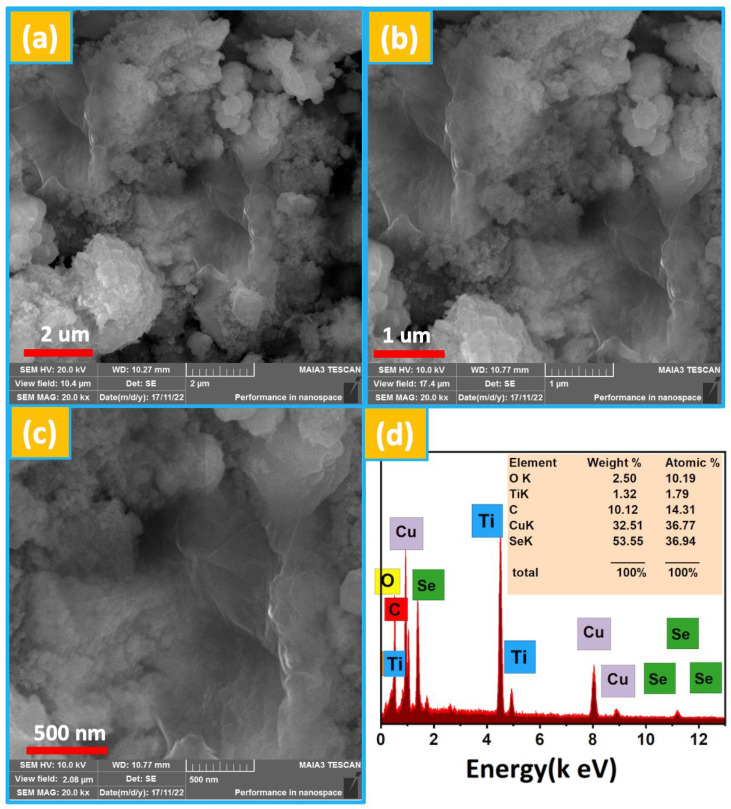
(**a**–**c**) Morphological analysis of the sample by the FESEM diagram at different scale bars, (**d**) EDX spectrum of the CuSe-TiO_2_-GO nanocomposite.

The surface overview of the composite was verified by field emission scanning electron microscopy (FESEM) analysis, and the related image is provided in Figure 3a–c with different scale bars. The FESEM diagram reveals that the CuSe is composed of a snow-crystal-like morphological appearance that provides sufficient pathways for the immobilization of ions during electrochemical activities. The CuSe is attached to the TiO_2_ and wrapped with GO. The good overlapping of the CuSe and TiO_2_ reduced the volume variation after inserting K-ions into the host matrix. It enhanced the overall capacitance due to its excellent conductivity. Moreover, the GO offers conductive pathways for charge kinetics. The collective contribution of different metal cations and carbon materials boosts the final performance of the electrode, which can be validated from the electrochemical performance. Additionally, no other impurity residues were absent on the surface of the CuSe-TiO_2_-GO ternary nanocomposite, revealing the high purity of the sample. This can be further verified by EDX analysis, as given in Figure 3d. As shown in Figure 3d, only respective elements can be distinguished in the spectrum, e.g., O, Cu, Se, Ti, and C peaks were detected, proving the samples’ high purity, which coincides with the FESEM and X-ray diffraction investigations.

**Figure 4:**
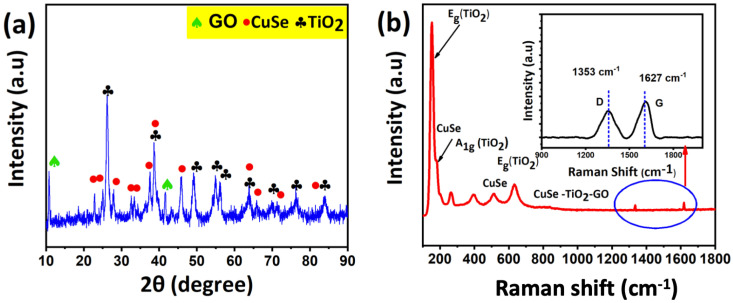
(**a**) X-ray diffraction pattern and (**b**) Raman analysis. The inset is the magnified view of the D and G bands of GO in the CuSe-TiO_2_-GO nanocomposite.

The CuSe-TiO_2_-GO ternary nanocomposite phase purity, adequate formation, and crystallinity were confirmed by X-ray diffraction analysis, and the results are given in Figure 4a. The X-ray diffraction pattern of the CuSe-TiO_2_-GO nanocomposite showed a collective appearance of CuSe, TiO_2_, and GO in the final product, showing the appropriate synthesis of the product—more specifically, the TiO_2_ tetragonal anatase crystal phase with the JCPDS No: 01-084-1285 relative to the 2θ values at 25.30°, 38.5°, 48.03°, 53.8°, 55°, 62.69°, 68.76°, 70.2°, 75.05°, and 82.76° assigned to (101), (112), (200), (1050, (211), (204), (116), (220), (215), and (224) planes, as depicted in Figure 4a. Additionally, the CuSe sample also showed a signature in the X-ray diffractogram with a peak at 26.2, 31.7°, 32.6°, 45.5°, 53.3°, 57.1°, and 65.2°, related to the miller indices of (100), (001), (120), (110), (200), (111), (002), and (208), which could be oriented as a hexagonal crystal structure, which coincides nicely with the JCPDS#00-027-0185. Moreover, the GO peaks at a 2θ value of 10.4°, corresponding to (001) and 43.4° (111) crystalline plane values. After that, no other impurities/byproducts/residues were absent during the formation process of the samples, marking the high purity, and the sharp peaks indicate the product’s excellent crystallinity.

The low-temperature Raman analysis can further validate this, and their corresponding graph is schematically shown in Figure 4b. Raman shifts at 150 cm^−1^ (Eg), 410 cm^−1^ (A_1g_), 520 cm^−1^ (B_1g_), and 620 cm^−1^ (E_g_) are the vibrational modes of the TiO_2_. Additionally, the CuSe sample displayed three vibrational modes at 209.3 cm^−1^ (A_1g_), 256.4 cm^−1^, and 492.7 cm^−1^ (E_g_), which could be assigned to the vibrational stretching mode (A_1g_) due to the Cu-Se bond. Two new peaks are established at 1353 cm^−1^ due to the D and G bands around 1627 cm^−1^ of the GO, as shown in the zoomed region in the inset in Figure 4b. The Raman analysis is also consistent with the X-ray diffraction results, which leads to the successful formation of the potential CuSe-TiO_2_-GO ternary nanocomposite.

**Figure 5:**
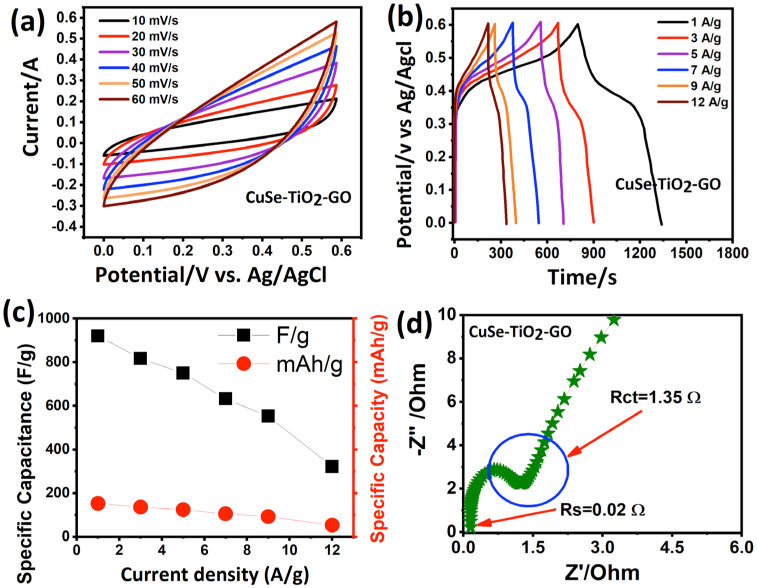
The CuSe-TiO_2_-GO nanocomposite and its capacitive performance analysis, (**a**) CV profile, (**b**) discharge/charge platforms, (**c**) variation in capacitance with current rates, (**d**) impedance plot.

The electrochemical properties of the CuSe-TiO_2_-GO ternary nanocomposite electrode were carefully judged by several electrochemical testing techniques utilizing an aqueous conductive medium. Generally, a three-electrode setup was operated to determine the host electrode’s capacitive charge storage signature, reversible/irreversible reactions, and rate capability. Convincingly, the cyclic voltammogram of the CuSe-TiO_2_-GO ternary nanocomposite electrode is displayed in Figure 5a between 0.0 and 0.6 V as the potential range with various scans. It was seen that a mirror voltammogram was well sustained from the low scan speed to higher scans, demonstrating the increased accessibility of the ions during the electrochemical process. The enclosed loop area expands with changing scan speeds, indicating the excellent reversibility and rate capability of the CuSe-TiO_2_-GO ternary nanocomposite electrode. Due to the high resistance and polarization phenomena, more negative-to-positive potentials were effectively seen in the voltammograms [34,42]. The discharge/charge platforms of the CuSe-TiO_2_-GO ternary nanocomposite electrode were performed utilizing various currents. Their corresponding plot is depicted in Figure 5b. The discharge platforms consist of three parts, a sudden voltage drop due to the internal resistance, a curved region denoting the faradaic reactions due to the pseudocapacitive nature of the CuSe and TiO_2_, and, lastly, the double-layer contribution from GO. The well-defined shape analogy from minor to more effective current rates sounds towards the high speed and reversibility of the composite electrode. Due to the distinguished voltage plateaus, the discharge/charge platforms demonstrated the pseudocapacitive response. Based on Equation (1), the capacitance at the desired currents is further calculated, and the results are plotted against different current rates in Figure 5c.

C = I × t/V × m(1)

V, m, and t specify the potential, the mass loading on the current collector, and the discharge time, while I denotes the current enclosed by the CV.

A high 920 F/g (153 mAh/g) is calculated for the CuSe-TiO_2_-GO ternary nanocomposite electrode at 1 A/g, which is much larger than that in the previous literature, such as 543.9 F/g for CuSe@FeOOH [35], 370 F/g (CuSe@TiO_2_) [34], 165 F/g (graphene@TiO_2_) [43], 837.7 F/g for Poly (methyl methacrylate, PMMA)/GO/IrO_2_ [44], 895 F/g for Cr_2_O_3_/GO/polyaniline (PANI), 280 F/g for PANI-H_2_SO_4_, TiO_2_ [45], NiS/GO (800 F/g) [46], 743 F/g for the GO/PANI/Ni(OH)_2_ nanocomposite [47], indium tin oxide (ITO)/GO/VO composite of 949.6 F/g [48], 773 F/g for MnS/GO/PANI [49], 251 F/g for GO@ multiwalled carbon nanotubes (MWCNTs) [50], 707 F/g for MWCNT/GO/NiCo_2_O_4_ hybrid composite [51], and 355.2 F/g for the PANI-GO [52] composite, respectively, as shown in Figure 5c. Table 1 illustrates the capacitance values of the CuSe-TiO_2_-GO ternary nanocomposite at the desired current rates. The only outer surface interaction was observed at the electrode/electrolyte interface for the CuSe-TiO_2_-GO ternary nanocomposite electrode at more significant current rates; hence, a continuous decay in capacitance was seen. Coulombic efficiency is also calculated, as tabulated in Table 1. It was attractive to note that the coulombic efficiency first decreased due to a low discharge time and then increased and sustained. The poor coulombic efficiency is due to the parasitic reaction during discharge cycles, leading to the poor Coulombic efficiency of the active electrode. The impedance spectroscopy (IS) further deeply analyzes the charge transport kinetics in an aqueous medium regarding charge transfer and solvent resistance, as shown in Figure 5d. Generally, the IS shines a light on the kinetics of the charge transfer process in an electrochemical system. The steep vertical line denotes the diffusion resistance of the electrolyte, the intersection at the real axis defines the solution resistance, and the half-circle with its diameter signifies the charge transfer resistance of the host electrodes. Herein, our work manifests the smallest values of the resistances (see Figure 5d), supporting the high conductivity of the CuSe-TiO_2_-GO nanocomposite electrode, which confirms the prompt supply of the ions to the inner and outer surfaces of the electrode. Additionally, the multicomponent in the composite matrix brings synergy, which promotes effective utilization, and structural integration is sustained during repeated electrochemical discharge and charge processes.

**Figure 6:**
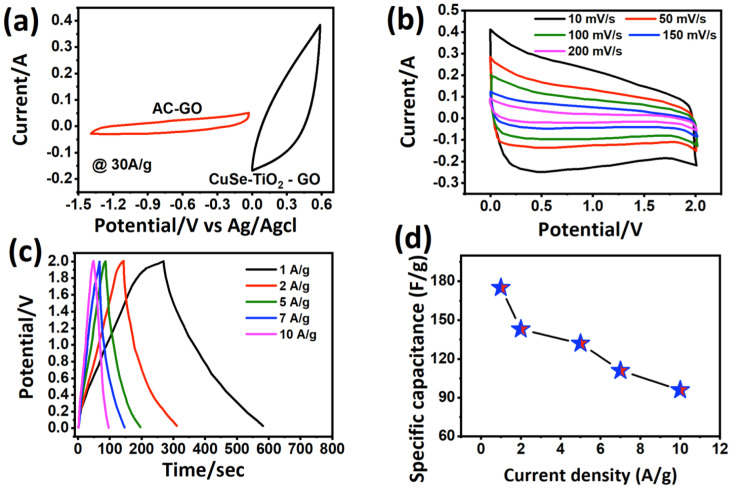
The energy storage performance of the CuSe-TiO_2_-GO||AC-GO||KOH hybrid supercapacitor, (**a**) CV profile from 0 to 2 V with several scans, (**b**) discharge/charge platforms, (**c**) variation in capacitance with the current rate, (**d**) specific capacitance versus current density.

The electric charge storage performance of the CuSe-TiO_2_-GO ternary nanocomposite was progressively assessed in a 3 M KOH aqueous solution as a conducting medium for K-ion migration in an electrolyte towards the negative electrode and OH^−^ ions to the cathode during the charging process. An electron from the external circuit moves toward the anode to maintain charge neutrality. Theoretically, the approximate upper cutoff voltage is expected to be as high as 2 V (adding the positive and negative voltages). The CV diagram of the fabricated CuSe-TiO_2_-GO||AC-GO||KOH hybrid supercapacitor is shown in Figure 6a from 0 to 2 V, utilizing the CuSe-TiO_2_-GO nanocomposite and the activated carbon with GO (AC-GO) as the cathode and anode electrodes, respectively. The CV diagram was taken at disparate scans ranging from 10 to 50, 100, 150, and 200 mV/s (see Figure 6b). No apparent change in the enclosed loops and analog shape was sustained in all the scanning, revealing the excellent rate capability and superior power delivery with good reversibility. The CV diagram displayed a non-rectangular shape owing to the pseudocapacitive charge storage mechanism of the CuSe-TiO_2_-GO||AC-GO||KOH hybrid supercapacitor. The absence of a CV tail towards the maximum voltage cutoff signifies not much electrolyte decomposition, possibly due to the expansion of OER/HER activities. Meanwhile, this further confirms that the chosen voltage is the stable and suitable voltage limit for the as-built CuSe-TiO_2_-GO||AC-GO||KOH hybrid supercapacitor. To support our results more convincingly, continuous discharge/charge platforms were performed at different currents, as depicted in Figure 6c. It is noted that the upper voltage cutoff (herein, 2 V) is preserved at numerous current rates. The discharge time equates to the charging time, indicating the tremendous coulombic efficiency of the CuSe-TiO_2_-GO||AC-GO||KOH hybrid supercapacitor. The shape of the discharge curves is unchanged, as designated by the high-rate performance of the CuSe-TiO_2_-GO||AC-GO||KOH hybrid supercapacitor. According to Equation (1), the capacitance for the CuSe-TiO_2_-GO||AC-GO||KOH hybrid supercapacitor was determined, as displayed in Figure 6c. It was worth noting that a significantly large capacitance of 175 F/g and 96 F/g was maintained when the current prolonged from 1 to 10 A/g, indicating a high capability (54.8%) of the CuSe-TiO_2_-GO||AC-GO||KOH hybrid supercapacitor, as depicted in Figure 6d. The acquired capacitance of the CuSe-TiO_2_-GO||AC-GO||KOH hybrid supercapacitor surpasses previous literature reports: 103.4 F/g GO-Ppy-Ag//AC [53], 152 C/g CoMoO_4_@r-GO||AC [54], 189 F/g for Fe_2_O_3_@GO//Ni_3_(PO_4_)_2_@GO [55], 115.15 F/g for Poly(3,4-ethylenedioxythiophene, PEDOT) PEDOT/GO||AC [56], 152 F/g for PPy/Ni_2_P/GO//AC [49], and 100 F/g for Cr_2_O_3_/GO/Ppy||AC [57]. The distinct and detailed capacitance values at each current are listed in Table 2.

The two critical factors in estimating practical performance validation are specific energy and power. The following equations are utilized to obtain extraordinary specific energy/power [58].

Specific energy (S.E) = 1/7.2 CV^2^ (2)

Specific power (S.P) = 3600 E/t (3)

V, C, and t specify the CV and discharge platform voltage, capacitance, and discharge time.

**Figure 7:**
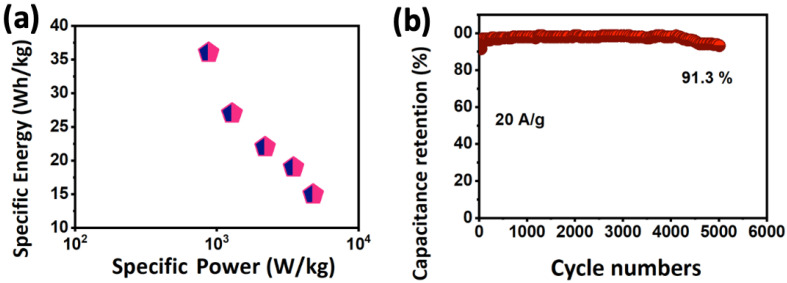
(**a**) Ragone plot, (**b**) cycling test in an aqueous solution.

According to Equation (2), a calculated specific energy of 36 Wh/kg was determined and reached its lowest point of 15 Wh/kg after 10 times increase in the current rates. The specific power of 4781 W/kg (Equation (3)) is displayed in Figure 7a. These values are comparable to or even higher than several literature reports, as summarized in Table 3. The specific power and energy at the designated current rates are schematically listed in Table 2. These high values of specific energy and power delivery confirmed the high conductivity of the heterostructured CuSe-TiO_2_-GO due to the synergistic impact of each component contributing to the enhanced performance of the ternary electrode material. Moreover, long-cycling stability is desirable for supercapacitors in real-life applications, and the related graph is shown in Figure 7b.

The longevity measurements are taken for 5000 cycles at the highest current rate to guarantee the synergy between multi-components (Figure 7b). A secure and stable longevity performance due to the high conductivity of the CuSe-TiO_2_-GO ternary composite was noticed, alleviating the volume change and accessing more accessible intercalation pathways for K-ions into the host electrode, resulting in a stable cycling performance of the CuSe-TiO_2_-GO||AC-GO||KOH hybrid supercapacitor. A slight decline was observed until 5000 cycles, which can be attributed to the structural degradation and morphological failure during fast discharge/charge cycles at an upsurge current. Overall, the CuSe-TiO_2_-GO||AC-GO||KOH hybrid supercapacitor achieved stable stability, with only 91.3% capacitance retained at such a considerable current, proving its promising feature for advanced energy storage devices.

## 4. Conclusions

This paper presents a simplistic and wet-chemical-assisted preparation of the CuSe-TiO_2_-GO ternary composite for the energy storage domain. The formation of the CuSe-TiO_2_-GO ternary composite was confirmed by the FESEM/EDX for morphological and elemental analysis, x-ray diffraction, and Raman investigations for structural and phase confirmation. At the same time, the charge storage performance was analyzed by electrochemical studies, such as impedance analysis, discharge/charge platforms, and CV analysis, respectively, in great detail. A high capacitance was executed due to the synergistic effect of the multi-component in the composite structure, which attained a high conductive backbone and supported fast charge kinetics. Notably, a voltage of 2 V was realized by the CuSe-TiO_2_-GO||AC-GO||KOH hybrid supercapacitor in an aqueous solution due to the expansion of HER/OER activities during electrolysis. Interestingly, a good specific energy of 36 Wh/kg was attained due to the high voltage and capacitance. A highly stable structural stability of 91.3% was realized during successive stability tests at an ultra-high current rate. Thus, our work recognizes the optimal voltage and capacitance, which enlarged the energy density of hybrid supercapacitors.

## Figures and Tables

**Table 1:** The current discharge rates and the capacitance values of the CuSe-TiO_2_-GO nanocomposite.

Current Density (A/g)	1	3	5	7	9	12
**Specific Capacitance (F/g)**	920	817	750	633	553	321
**Specific Capacity (mAh/g)**	153	136	125	105	93	53
**Coulombic Efficiency (%)**	69	58	54	77	73	73

**Table 2:** The capacitance and specific energy/power values at different current rates.

Current Density (A/g)	1	2	5	7	10
**Specific Capacitance (F/g)**	175	143	132	111	96
**Specific Energy (Wh/kg)**	36	27	22	19	15
**Specific Power (W/kg)**	875	1281	2193	3477	4781

**Table 3:** CuSe-TiO_2_-GO||AC-GO||KOH hybrid supercapacitor performance comparison with literature reports based on GO, CuSe, and TiO_2_ hybrid/asymmetric supercapacitors.

Electrode Material	Voltage (V)	Capacitance (F/g)	Specific Energy (Wh/kg)	Specific Power (W/kg)	Stability (%)	Ref.
**CoSe_2_/CuSe hybrid SC**	1.6	192.8	54.6	700	82.5@10k	[37]
**CuSe@TiO_2_||AC ASC**	1.8	70	31.5	4500	99.6@10k	[34]
**CuSe_2_/rGO||CuS ASC**	1.5	104	28.3	1538	86.5@5k	[59]
**TiO_2_/rGO||AC ASC**	3	89	42	800	80@10k	[60]
**Fe_2_O_3_@GO//Ni_3_(PO_4_)_2_@GO ASC**	1.6	189	67.2	1276.3	88@1k	[55]
**PEDOT/graphene oxide supercapacitor**	1.2	115.15	13.60	139.09	-	[56]
**CuS@carbon dot ASC**	1.4	103	28	700	90@5k	[61]
**2D/2D NiCo-MOF/GO ASC**	1.6	162 C/g	36.83	374.99	-	[62]
**Re-GO@NiS_2_ ASC**	1.6	80	28.31	800	83.34@10k	[63]
**MnSe/GO//AC**	1.6	56.25	31.25	6779.20	86.3@5k	[64]
**MnSe_2_/rGO//AC**	1.6	-	16.6	7200	99@10k	[65]
**CuSe-TiO_2_-GO||AC-GO||KOH hybrid supercapacitor**	**2**	**175**	**36**	**4781**	**91.3@5k**	**This work**

## Data Availability

Not applicable.
